# Prognostic impact of systolic blood pressure variability in people with diabetes

**DOI:** 10.1371/journal.pone.0194084

**Published:** 2018-04-11

**Authors:** Katy J. L. Bell, Lamiae Azizi, Peter M. Nilsson, Andrew Hayen, Les Irwig, Carl J. Östgren, Johan Sundröm

**Affiliations:** 1 Sydney School of Public Health, University of Sydney, Sydney, New South Wales, Australia; 2 School of Mathematics and Statistics, University of Sydney, Sydney, New South Wales, Australia; 3 Department of Clinical Sciences, Lund University, Malmo, University Hospital, Malmo, Sweden; 4 Australian Centre for Public and Population Health Research University of Technology Sydney (UTS), Sydney, New South Wales, Australia; 5 Department of Medical and Health Sciences, Linkoping University, Linkoping, Sweden; 6 Department of Medical Sciences, Uppsala University, Uppsala, Sweden; The University of Tokyo, JAPAN

## Abstract

**Objective:**

Blood pressure variability (BPV) has been associated with risk of cardiovascular events in observational studies, independently of mean BP levels. In states with higher autonomic imbalance, such as in diabetes, the importance of BP variability may theoretically be even greater. We aimed to investigate the incremental value of BPV for prediction of cardiovascular and all-cause mortality in patients with type 2 diabetes.

**Methods:**

We identified 9,855 patients without pre-existing cardiovascular disease who did not change BP-lowering treatment during the observation period from a Swedish primary health care cohort of patients with type 2 diabetes. BPV was summarized as the standard deviation (SD), coefficient of variation (CV), or variation independent of mean (VIM). Patients were followed for a median of 4 years and associations with cardiovascular and all-cause mortality were investigated using Cox proportional hazards models.

**Results:**

BPV was not associated with cardiovascular specific or all-cause mortality in the total sample. In patients who were not on BP-lowering drugs during the observation period (n = 2,949), variability measures were associated with all-cause mortality: hazard ratios were 1.05, 1.04 and 1.05 for 50% increases in SD, CV and VIM, respectively, adjusted for Framingham risk score risk factors, including mean BP. However, the addition of the variability measures in this subgroup only led to very minimal improvement in discrimination, indicating they may have limited clinical usefulness (change in C-statistic ranged from 0.000–0.003 in all models).

**Conclusions:**

Although BPV was independently associated with all-cause mortality in diabetes patients in primary care who did not have pre-existing cardiovascular disease or BP-lowering drugs, it may be of minimal clinical usefulness above and beyond that of other routinely measured predictors, including mean BP.

## Introduction

Within-person visit-to-visit variability of blood pressure has been demonstrated to be associated with risk of both stroke and coronary heart disease independently of mean blood pressure across clinic visits[1]. It is possible that this association is causal[2], and that blood pressure variability (BPV) may be an important entity especially in settings with higher autonomic imbalance[2], such as in diabetes. This is supported by some reports which found that visit-visit BPV was an independent predictor of both macrovascular[3–5] and microvascular disease[3] among people with type 2 diabetes, as well as all-cause mortality[5,6].

A recent systematic review of 41 cohort studies and clinical trials examining BPV and CVD, included 27 studies which measured BP variability in clinic measurements (including three of the diabetic studies cited above)[7]. Significant associations independent of mean BP were found with all-cause and CVD-specific mortality, but most studies were rated as at least moderate risk of bias. In addition to establishing strengths of the associations, the clinical value of these measures require investigation. We have previously found that additional measurements of clinic[8] or ambulatory blood pressure[9] were unlikely to be clinically useful for predicting cardiovascular disease even when they were independent predictors statistically.

Using a large sample of patients with type 2 diabetes in primary care, we aimed to investigate the magnitude of within-person visit-to-visit BPV; the associations of that variability with risk of cardiovascular and all-cause mortality; and the contribution of BPV to cardiovascular risk prediction above and beyond mean blood pressure and major cardiovascular risk factors in terms of overall model fit, discrimination, calibration and reclassification.

## Materials and methods

### Study sample

**Fig 1:**
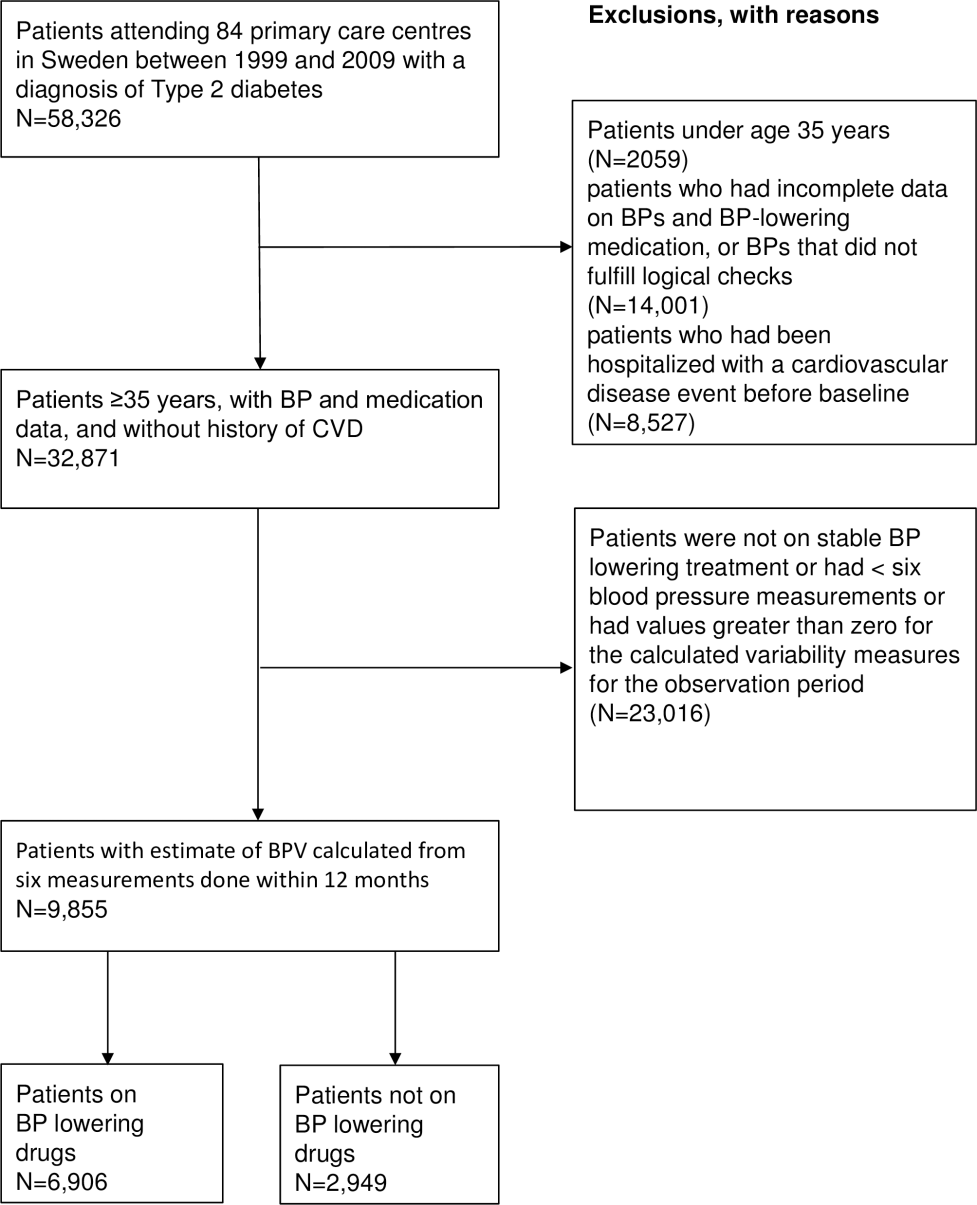
Flow of participants in the study. Selection of participants.

This observational study was based on patients with type 2 diabetes in Swedish primary care in the “Retrospective Epidemiological Study to Investigate Outcome and Mortality with Glucose Lowering Drug Treatment in Primary Care” (ROSE) study sample. Data were extracted in 2010 from electronic patient records from 84 primary care centres in Sweden by the Pygargus Customized eXtraction Program and the study has been described in detail previously[10,11]. The primary care centers were chosen to provide a good representation of Swedish primary care. The selection of participants for the current study is summarized in Fig 1. We included patients with type 2 diabetes who did not have CVD, who had at least six BP measurements on stable BP lowering treatment during the “observation period” (i.e. either off treatment or on same drug treatment regimen during the whole time the BPV was calculated), and who had values greater than zero for the calculated variability measures. We excluded patients under age 35 years, patients who had incomplete data on BPs and BP-lowering medication or BPs that did not fulfill logical checks, and patients who had values less than zero for the calculated variability measures. This left 9,855 patients included in main analysis of associations between BPV and mortality. As a sensitivity analysis, we also explored associations for the subpopulation of 2949 patients who were not taking BP lowering drugs during the observation period.

### Exposures

Blood pressure data were extracted from primary care electronic patient charts for the variables of systolic and diastolic BP. In Sweden, primary care BP reading is usually performed by public health nurses and is performed according to standardized methods, using the manual Korotkoff method or automatic measurements. Patients are typically told to avoid coffee and tobacco 30 min before the examination and conversation with the patient is normally not recommended during the procedure. BP is registered after 5 min rest in either the supine or sitting position, with an appropriate sized cuff. If several readings are performed, the calculated mean is recorded[11]. Home blood pressure measurements were not included in the study.

We calculated within-person visit-to-visit variability of systolic and diastolic blood pressure as the standard deviation (SD), coefficient of variation (SD/mean), and variation independent of mean (VIM)[1] of 6 consecutive measures made during the observation period (which varied in duration up to a maximum of 12 months). We constructed scatterplots and calculated correlation statistics between the three types of variability measure.

### Covariates

Age and sex were determined using the unique personal identification number allocated to all Swedish citizens. Measurements of total cholesterol, HDL cholesterol; smoking status, sex, BP lowering drug status, estimated glomerular filtration rate (eGFR), HbA1c, drugs to lower cholesterol and glucose, insulin, highest level of education, personal income, family income, marital status, and mean triglyceride level were extracted from primary care electronic patient charts as previously described[10]. Mean systolic or diastolic BP of the 6 consecutive measures were also included as covariates.

### Follow-up and outcomes

Follow-up time was from the last BP measurement in the observation period. The primary outcome was cardiovascular mortality (ICD-10 codes I00-I99) determined with high validity[12] by linkage to the Swedish national cause-of-death registry. The secondary outcome was death from any cause. Patients were followed until the first event of death, emigration, or December 31^st^, 2009.

### Ethical approval and trial registration

The study, which complied with the declaration of Helsinki, was approved by the Regional Ethical Review Board in Uppsala, Sweden. The ClinicalTrials.gov number is NCT 01121315. All data were fully anonymised before they were accessed.

### Statistical analysis

Associations between each of the variability measures with cardiovascular mortality and all-cause mortality were investigated using Cox proportional models. For the main results we explored linear associations by fitting the variability measures as continuous variables (on the log scale), and conducted sensitivity analyses to explore non-linear associations where we fitted the variability measures by quintiles and where we used penalised splines.

For the two samples (all patients without CVD and subpopulation who were not on BP lowering drugs) and three types of outcome (CVD mortality, all-cause mortality, and CVD mortality with competing risks), we built base models with (i) only risk variables included in the Framingham equation (age, systolic BP, total cholesterol, HDL cholesterol, smoking status, sex, BP lowering drug status), (ii) risk variables included in Framingham equation + other statistically significant predictors. Non-Framingham risk factors considered for inclusion were: estimated glomerular filtration rate (eGFR), HbA_1c_, drugs to lower cholesterol and glucose, insulin, highest level of education, personal income, family income, marital status, and mean triglyceride level. The mean of all measurements made on an individual during the observation period were used for the following risk factors: systolic and diastolic BP, total and HDL cholesterol; baseline measurements were used for all other potential risk factors. All continuous variables in the base models were log-transformed, for consistency with common CVD risk models such as Framingham[13].

We assessed the added value of the BPV measures above and beyond established CVD predictors, including mean BP. We did this by comparing the overall model fit (likelihood ratio tests) and discrimination (C-statistics) for the base models as outlined above, and for an equivalent model which included each BPV measure. For BPV measures where we found evidence of substantially improved discrimination, we planned to assess clinical usefulness by examining the effects on reclassification of people who did and did not die from CVD during follow-up[14]. As well as calculating hazard ratios for the BPV measures on the log scale, to improve interpretation, we also estimated hazard ratios for 25 and 50 percentage increases in each measure. We did this by applying a log transformation to the percent increase, multiplying by the relevant beta coefficient and then back-transforming the result. For example the hazard ratio for a 25% increase in a BPV measure was calculated using the following formula: HR = exp(β*log(1.25)).

Proportional hazards assumptions were assessed by inspecting Schoenfeld residuals and cumulative incidence curves. Multiplicative interaction terms between BPV measures and gender, age, mean blood pressures, antihypertensive treatment, body mass index, HbA_1c_, and eGFR were also investigated.

## Results

**Fig 2:**
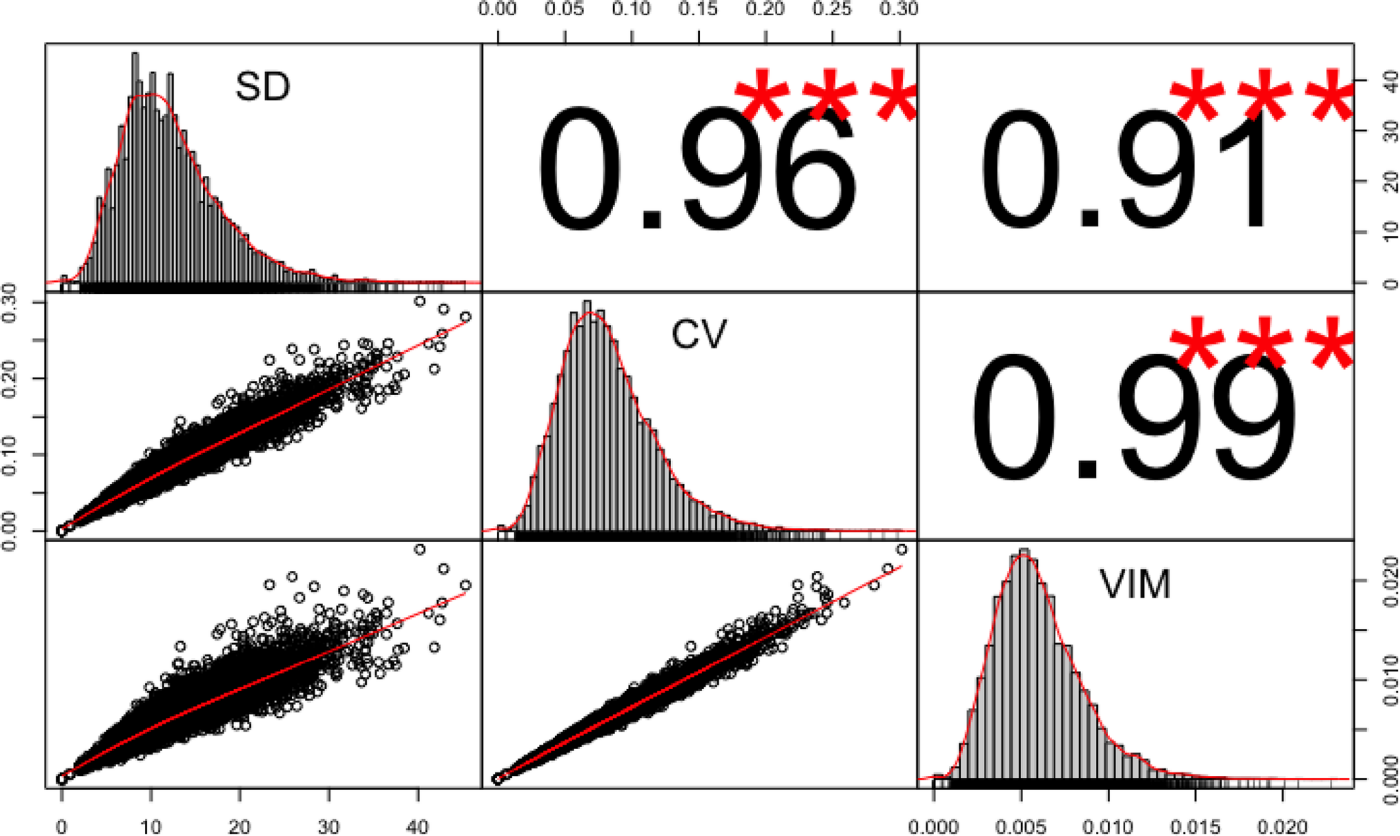
Distribution plots, bivariate scatterplots and correlation coeffficient for SD, CV and VIM (natural scale before log transformation). Distributions for SD, CV and VIM are shown in the diagonal cells of the figure. Bivariate scatterplots with a fitted line are shown in the lower left of the figure: SD and CV (middle left cell), SD and VIM (lower left cell) and CV and VIM (lower middle cell). Three different axes are presented corresponding to the variability measures: SD ranges from 0 to 45 mmHg, CV range from 0 to 0.30% and VIM ranges from 0 to 0.023. Pearson correlation values are shown in the upper right of the figure and marked by ***: SD and CV (0.96, upper middle cell), SD and VIM (0.91, upper right cell) and CV and VIM (0.99, middle right cell).

Summary data on the three types of BPV measures for the study population without CVD at baseline are presented in Fig 2 (SD, CV and VIM). All three variability measures showed a leftward skewed distribution before log transformation. There was a very high correlation between the three measures, with correlation coefficients of 0.91 to 0.99. Similar results were found for the variability measures for the subpopulation who were not on BP lowering drugs. Summary characteristics of all Framingham risk variables and other statistically significant variables are shown in Table 1. Compared with the full study population, those who were not on BP lowering drugs were younger and had lower systolic BP, but were at higher risk from all other risk factors.

**Table 1:** Characteristics of 9855 diabetic patients without CVD^1^ at baseline.

	All patients n = 9855		Patients not on BP lowering drugsn = 2949	
	*Mean (SD) or %*		*Mean (SD) or %*	
Age (years)	63.8	(10.7)	60.1	(11.4)
Total Cholesterol (mM/L)	5.3	(1.0)	5.4	(1.0)
HDL Cholesterol (mM/L)^2^	1.5	(0.8)	1.4	(0.8)
Sex (Female)	47.4%		43.6%	
Smoker	16.6%		20.2%	
Systolic BP (mmHg)^3^	146.0	(17.3)	135.8	(13.9)
BP lowering drug use^3^	70.1%		0%	
Estimated Glomerular Filtration Rate (eGFR) (mL/min/1.73m^2^)	80.0	(17.7)	84.9	(16.9)
HbA1c (%)^4^	7.1	(1.2)	7.3	(1.2)

Footnotes

1. CVD = cardiovascular disease

2. HDL = high density lipoprotein

3. BP = blood pressure

4. HbA1c = haemoglobin A1c

During a median follow-up of 4 years (range 1 to 11 years), 1856 people died, of whom 1489 died of cardiovascular disease. The results for added predictive value of the variability measures in all patients without a history of CVD are presented in Table 2. The adjusted hazard ratios were all close to 1.0 for all three outcomes (p-values all >0.20). There was no evidence that any of the variability measures added to the predictive value of either the Framingham risk factors alone, or in combination with other significant predictors (eGFR and HbA_1c_), and there was no improvement in discrimination (change in C-statistic 0.000 to 0.001). Results remained unchanged when the variability measures were fitted as quintiles and when penalized splines were used. We did not find any significant interactions between BPV measures and gender, age, mean blood pressures, antihypertensive treatment, body mass index, HbA_1c_, and eGFR.

**Table 2:** Incremental value of variability measures estimated in Cox models for all diabetic patients without CVD at baseline^1^.

		CVD Mortality^1^		CVD Mortality^1^, competing risks		All-cause Mortality	
Variability Measure		FRS Variables^2^	FRS Variables Plus^3^	FRS Variables^2^	FRS Variables Plus^4^	FRS Variables^2^	FRS Variables Plus^3^
**SD**	HR (log scale)	1.01 (0.89–1.15)	1.02 (0.89–1.16)	1.09 (0.93–1.29)	1.09 (0.93–1.30)	1.01 (0.90–1.13)	1.02 (0.90–1.14)
	HR (25% increase on natural scale)^5^	1.00	1.00	1.01	1.01	1.00	1.00
	HR (50% increase on natural scale)^5^	1.00	1.00	1.02	1.02	1.00	1.00
	∆c	0.000	0.000	0.001	0.001	0.000	0.000
	p-value	0.87	0.80	0.27	0.29	0.93	0.80
**CV**	HR (log scale)	0.99 (0.86–1.14)	1.00 (0.87–1.15)	1.10 (0.93–1.30)	1.10 (0.92–1.30)	0.99 (0.87–1.12)	0.99 (0.87–1.12)
	HR (25% increase on natural scale)^5^	1.00	1.00	1.01	1.01	1.00	1.00
	HR (50% increase on natural scale)^5^	1.00	1.00	1.02	1.02	1.00	1.00
	∆c	0.000	0.000	0.001	0.001	0.000	0.000
	p-value	0.90	0.99	0.28	0.29	0.83	0.83
**VIM**	HR (log scale)	0.98 (0.85–1.13)	0.99 (0.86–1.14)	1.10 (0.92–1.30)	1.09 (0.92–1.30)	0.98 (0.86–1.11)	0.98 (0.86–1.11)
	HR (25% increase on natural scale)^5^	1.00	1.00	1.01	1.01	1.00	1.00
	HR (50% increase on natural scale)^5^	1.00	1.00	1.02	1.02	1.00	1.00
	∆c	0.000	0.000	0.001	0.001	0.000	0.000
	p-value	0.78	0.87	0.29	0.30	0.71	0.71

Footnotes

1. CVD = cardiovascular disease

2. FRS Variables = Framingham Risk Score variables: age, gender, smoking status, diabetes, systolic BP, BP lowering drug

3. FRS Variables Plus, CVD mortality and All-cause mortality models = Framingham Risk Score variables + HbA1c, eGFR, Insulin

4. FRS Variables Plus, CVD Mortality, competing risks model = Framingham Risk Score variables + HbA1c, Insulin, lipid lowering drugs

5. Percentage increment is for each variability measure where all other variables remain constant. For interpretation of percentage increase, please see Results, Estimates of effect of risk factors.

The results for added predictive value of the variability measures in the subpopulation who were not taking BP lowering drugs, are presented in Table 3. The adjusted hazard ratios for the variability measures for CVD-specific mortality were not statistically significant and ranged from 1.00 to 1.04 per 50% increase in each variability measure on the natural scale (HRs ranged from 1.00 to 1.23 on the log-scale; all confidence intervals all included 1 and p values all >0.10). There were negligible improvements in discrimination (change in C-statistic ranged from 0.001 to 0.002). The adjusted hazard ratios for CVD-specific mortality allowing for competing risks from other causes of death were all close to 1 (p values all >0.50) and there were no improvements in discrimination (change in C-statistic all 0.000). However, for all-cause mortality, the adjusted hazard ratios were statistically significant and ranged from 1.04 to 1.05 per 50% increase in each variability measure on the natural scale (HRs ranged from 1.10 to 1.32 on the log-scale), with associated p-values <0.05. For example a diabetic person with a within-person SD for their systolic blood pressure of 15mmHg would have approximately 1.05 times higher risk of premature death compared with a person with a within-person SD of 10 mmHg. We found only minimal improvements in discrimination (change in C-statistic 0.001 to 0.003).

**Table 3:** Incremental value of variability measures estimated in Cox models for patients without history of CVD^1^, and who were not on BP lowering drugs.

		CVD Mortality^1^		CVD Mortality^1^, competing risks		All-cause Mortality	
Variability Measure		FRS Variables^2^	FRS Variables Plus^3^	FRS Variables^2^	FRS Variables Plus^4^	FRS Variables^2^	FRS Variables Plus^3^
**SD**	HR (log scale)	1.24 (0.95–1.63)	1.20 (0.92–1.58)	0.93(0.69–1.23)	0.93 (0.70–1.23)	1.34 (1.06–1.70)	1.30 (1.03–1.65)
	HR (25% increase on natural scale)^5^	1.00	1.02	0.99	0.99	1.03	1.03
	HR (50% increase on natural scale)^5^	1.00	1.03	0.99	0.99	1.05	1.05
	∆c	0.002	0.002	0.000	0.000	0.003	0.002
	p-value	0.13	0.20	0.60	0.61	0.02	0.03
**CV**	HR (log scale)	1.15 (0.89–1.49)	1.11 (0.86–1.45)	0.97 (0.73–1.29)	0.98 (0.73–1.30)	1.28 (1.06–1.70)	1.23 (0.98–1.55)
	HR (25% increase on natural scale)^5^	1.01	1.01	1.00	1.00	1.02	1.02
	HR (50% increase on natural scale)^5^	1.02	1.02	1.00	1.00	1.04	1.04
	∆c	0.001	0.001	0.000	0.000	0.003	0.001
	p-value	0.29	0.47	0.83	0.86	0.04	0.09
**VIM**	HR (log scale)	1.23 (0.94–1.60)	1.20 (0.92–1.57)	0.96 (0.71–1.28)	0.96 (0.72–1.28)	1.33 (1.06–1.69)	1.30 (1.03–1.64)
	HR (25% increase on natural scale)^5^	1.02	1.02	1.00	1.00	1.03	1.03
	HR (50% increase on natural scale)^5^	1.04	1.03	0.99	0.99	1.05	1.05
	∆c	0.002	0.002	0.000	0.000	0.003	0.002
	p-value	0.13	0.22	0.76	0.78	0.02	0.04

Footnotes

1. CVD = cardiovascular disease

2. FRS Variables = Framingham Risk Score variables: age, gender, smoking status, diabetes, systolic BP, BP lowering drug

3. FRS Variables Plus, CVD mortality and All-cause mortality models = Framingham Risk Score variables + HbA1c, eGFR, Insulin

4. FRS Variables Plus, CVD Mortality, competing risks model = Framingham Risk Score variables + HbA1c, Insulin, lipid lowering drugs

5. Percentage increment is for each variability measure where all other variables remain constant. For interpretation of percentage increase, please see Results, Estimates of effect of risk factors.

Fitting the variability measures as quintiles and using penalized splines resulted in similar findings. Likewise the models for diastolic blood pressure variation also gave similar results.

## Discussion

In this large cohort study of people with type 2 diabetes, we found no evidence that BP variability was independently associated with CVD-specific or all-cause mortality in the total sample of patients without pre-existing CVD. However, in a subgroup of patients who were not on BP-lowering drugs, variability measures were statistically associated with all-cause mortality. The variability measures only gave very minimal improvement in discrimination when added to established risk factors in this subgroup. This indicates that although BPV may result in detectable improvements in mortality prediction, these are unlikely to translate into clinical usefulness for risk prediction, above and beyond that of other routinely measured predictors.

Strengths of the study include the large number of patients, zero loss to follow-up, and high quality of the data linkage to mortality outcomes in national registers. To avoid confounding by changes in treatment (which may reflect less well controlled mean BP), we restricted our study population to individuals who were not on BP lowering treatment, or on a stable regimen during the observation period which determined BPV. Our statistical analysis was rigorous and thorough, and we adjusted for a number of known confounders. Weaknesses include the observational nature of the study, and the fact that the study was retrospective and used routinely collected data. We did not have information on the time of day that individual blood pressure measurements were made, nor the season. The observation period for the six BP measurements used to calculate BPV varied in duration up to 12 months. The use of different BP measurement devices on different occasions may have introduced random noise into our estimates of BPV. However as this is more likely to affect between patient BPV rather than within patient BPV (if the same type of device was used for a patient attending the same clinic), then this is unlikely to have masked any true associations with CVD. Moreover, the method sometimes used for BP measurement in Swedish primary care whereby the calculated mean is recorded if several readings are performed, will tend to decrease the component of BPV due to random measurement variability. BPV so estimated may more accurately reflect natural biological BP variability. A high proportion of our study population were on BP-lowering drugs, and this is likely to “dampen” natural BP variability which may obscure potential associations with CVD outcomes. The analysis limited to individuals who are not on these medications may be a better reflection of the potential associations. The added value of the variability measures was to models that included Framingham risk factors, but using coefficients derived in this data set. If standard Framingham risk estimation was done using published risk scores used in clinical practice[13,15], the added value of the variability measures may have been greater.

Our study is larger than some studies[3,4,6,16] but considerably smaller than other studies[5,17] of type 2 diabetes patients which have found positive associations between SBP variability and CVD outcomes, including mortality. As far as we are aware, none of these other studies attempted to restrict analysis to patients who were either off BP lowering treatment or on the same drug treatment regimen during the whole time the blood pressure variability was calculated. Failure to do so could mean that BP variability is actually operating as a marker for increases in BP treatment because of poorly controlled mean BP, which is the true risk factor. We found clearly skewed distributions for all the variability measures, and so applied log transformations before adding to the models. However it appears that most if not all other studies have not done this, possibly accounting for some of the differences found between ours and others’ studies.

The association between BPV and all-cause mortality for patients with diabetes (where this is not “dampened” by BP lowering medication) may be a result of increased arterial stiffness in these subjects. That is, BP variability may be a marker of age-related arterial morphological changes which result in arterial stiffness[18]. Arterial stiffness (as measured by pulse wave velocity) occurs at increased rates in patients with type 2 diabetes, and has been found to predict not only CVD mortality, but also all-cause mortality[19]. Increased arterial stiffness may itself be a marker of vascular ageing and biological ageing in general. Thus, our finding of an association between BP variability and all-cause mortality in patients with diabetes who are not on BP lowering drugs may be as a result of accelerated vascular aging in these subjects. Another possibility is that an increased BPV in persons with diabetes is a sign of autonomic dysfunction, in the form of impaired baroreflex sensitivity[20]. Cardiovascular autonomic neuropathy is a serious complication of diabetes, with an estimated doubling of mortality risk compared to absence of this neuropathy[21]. Both of these explanations are linked to increased duration, or target organ damage, of diabetes. We had no reliable data on duration of diabetes in this study.

Despite these findings suggesting BP variability may be a marker of factors which cause premature death in patients with diabetes, we found that there was limited clinical usefulness in terms of risk prediction beyond established risk factors. In all of the models, we found that mean BP level was a more important predictor of CVD-mortality and all-cause mortality than variability.

Other, more direct, markers of vascular ageing or autonomic neuropathy may be more clinically useful for risk prediction in type 2 diabetes than BP variability, and searching for them may be fruitful.
