# Correction: Prognostic impact of systolic blood pressure variability in people with diabetes

**DOI:** 10.1371/journal.pone.0224538

**Published:** 2019-10-24

**Authors:** Katy J. L. Bell, Lamiae Azizi, Peter M. Nilsson, Andrew Hayen, Les Irwig, Carl J. Östgren, Johan Sundström

The seventh author's name is spelled incorrectly. The correct name is: Johan Sundström.
